# Supplementary material for: Inference is bliss: Simulation for power estimation for an observational study of a cholera outbreak intervention
Source: PLoS Negl Trop Dis. 2022 Feb 16;16(2):e0010163. doi: 10.1371/journal.pntd.0010163 (PMC8887757; doi:10.1371/journal.pntd.0010163)

**Technical Appendix for *Simulation for power estimation for an observational study of a cholera outbreak intervention***

1. **Model fitting**

The plot of standardized residuals versus fitted values was skewed left, showing some independence of the residuals from the fitted value and unequal variance. The random effect for the delay to detection of the index case, did not appear to substantially improve on the fit of the GLMM on the GLM, as the marginal R-squared (R^2^_m_=0.12, which represents the variance of fixed effects) was similar to the conditional R-squared (R^2^_c_=0.13, which represents the variance of fixed and random effects). This may have resulted from the simulated delay to detection of the index case failing to capture a realistic gradient in surveillance system sensitivity. Accordingly, little variance among the random effect levels was shown in the plot of fitted random effect values versus residuals. The GLMM and GLM (without random effects) produced similar effect sizes and variance.

1. **Sensitivity analyses**

Power estimates are shown in Table A and sensitivity analyses are visualized in Figures A—D.

|  |  |  |  | **Number of rings** | | | | | |
| --- | --- | --- | --- | --- | --- | --- | --- | --- | --- |
| ***R_0_*** | **D** | **Duration** | **Coverage** | **50** | **75** | **80** | **100** | **125** | **150** |
| 2 | 1.5 | 1 | 80% | 52.4 | 71.7 | 73.7 | 80.6 | 88.7 | 94.7 |
| 2 | 1 | 1 | 80% | 57.3 | 77.1 | 81.2 | 85.8 | 92.7 | 96.2 |
| 1.5 | 1.5 | 1 | 80% | 33.6 | 37.4 | 49.5 | 49.7 | 58.3 | 62.8 |
| 2 | 1.5 | 2 | 80% | 44.4 | 60.4 | 60.5 | 69.5 | 78.7 | 84.7 |
| 2 | 1.5 | 1 | 50% | 52.9 | 64.2 | 68.9 | 77.5 | 85.9 | 92.4 |
| 2 | 1.5 | 1 | 60% | 51.3 | 64.6 | 70.6 | 76.8 | 85.3 | 92.3 |
| 2 | 1.5 | 1 | 75% | 53.6 | 68.0 | 72.8 | 79.1 | 84.7 | 92.1 |

**Table A.** Power estimates from main simulations and sensitivity analyses. Shading indicates the variable that was changed (grey), and where power estimates were farthest from the 80% target (≤69%, in orange), close to the target (≥70 to 79%, in light green), and at or above the target (≥80%, in dark green).

**Fig A**: power estimates using a duration of implementation of two days


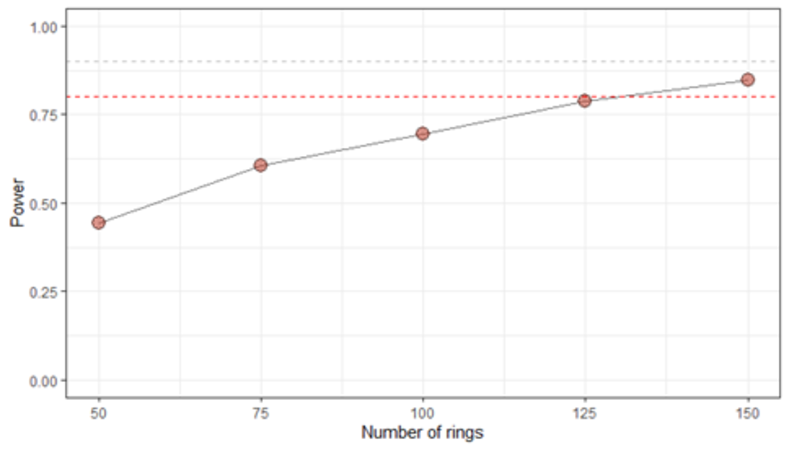


**Fig B**: power estimates using a population coverage of 50%


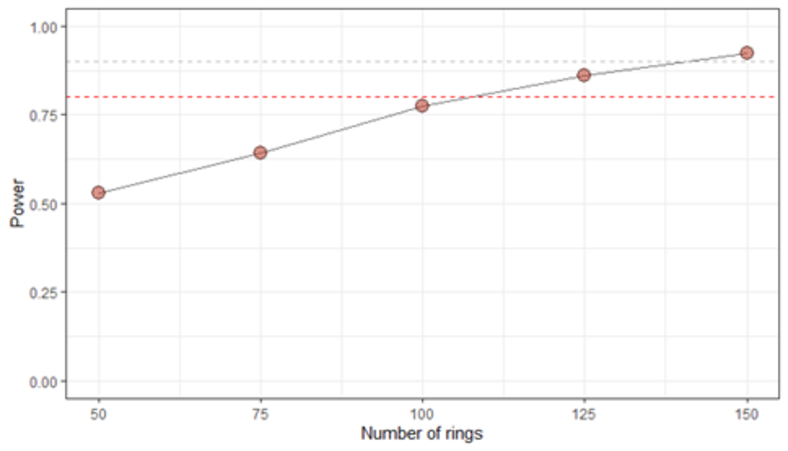


**Fig C**: power estimates using a population coverage of 60%


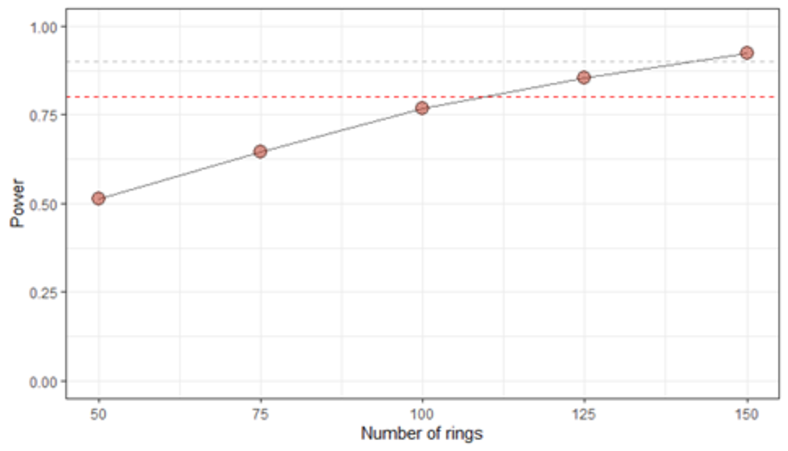


**Fig D**: power estimates using a population coverage of 75%


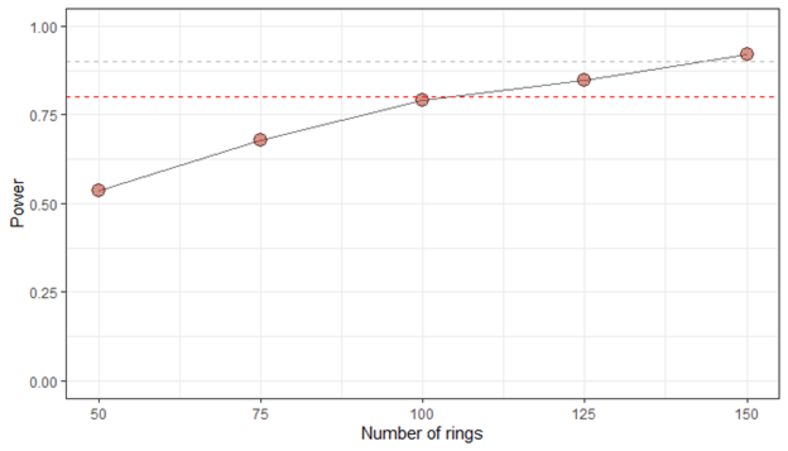

Supplement: S1 Text — Table A. Power estimates from main simulations and sensitivity analyses Fig A: power estimates using a duration of implementation of two days Fig B: power estimates using a population coverage of 50% Fig C: power estimates using a population coverage of 60% Fig D: power estimates using a population coverage of 75%. (DOCX) [file pntd.0010163.s001.docx]
