# Supplementary material for: Inference is bliss: Simulation for power estimation for an observational study of a cholera outbreak intervention
Source: PLoS Negl Trop Dis. 2022 Feb 16;16(2):e0010163. doi: 10.1371/journal.pntd.0010163 (PMC8887757; doi:10.1371/journal.pntd.0010163)
Supplement: Attachment — Submitted filename: Cover letter_R1.docx [file pntd.0010163.s002.docx]

December 9, 2021

**Manuscript ID:** PNTD-D-21-01219

**Title:** Inference is bliss: Simulation for power estimation of a cholera outbreak intervention study (short report)

Dear Dr. Yang,

Thank you to the editor and reviewers for reviewing our paper and providing helpful critiques to improve it further. Please find below a point-by-point response for each comment.

We hope you consider our revision favorably.

Ruwan Ratnayake, Corresponding author

**Reviewer 1 comments:**

1. **Only a generalized linear mixed model (GLMM) was used to estimate the response variable, it always makes good simulations and inferences. But I was also curious whether the finding showed similar results under different simulation models. Due to different scenarios and the habits of researchers, the conclusions obtained in the GLMM model are not necessarily applicable to guide other models; For example, under more powerful simulation techniques such as popular machine learning models, whether the need for sample sizes will decrease?**

Thank you for this suggestion to explore other modelling methods. We believe the reviewer is asking about whether the results hold if a machine learning model was used for the study analysis, instead of GLMM. We would like to clarify that GLMM was selected as the analytical method as the prospective observational study design includes clusters. GLMM takes the clustered nature of the data into account and calculates the variance correctly. GLMM also gives an effect estimate, which we require in this study to judge the direction and magnitude of CATI’s effect on cholera incidence. Other modelling methods that account for clustering include generalized estimating equations (GEE) and generalized additive models (GAM).

We considered whether any machine learning methods would be of use for this scenario. We concluded that machine learning methods would not produce an effect estimate and would instead tell us about the relative importance of covariates in the regression (i.e., random forest, neural net), although we have only a single exposure variable. They could predict the incidence (i.e., random forest) but we only require an effect estimate. They could perform regularization and deal with overfitting (i.e., Lasso and Ridge regression) but we would need more data to benefit from these methods.

Therefore, we did not comment further on machine learning methods. However, we added the following text to the methods section to justify our choice of GLMM, as compared to GEE and GAM, for readers who may be interested in model choices:

**Page 12-3 [lines 246-257]:** As the health of individuals in the same ring may be correlated, only regression modeling approaches that account for the clustered nature of the data can be used for the study analysis. This includes GLMM, generalized estimating equations (GEE) and generalized additive models (GAM). GLMM uses random effects to account for contextual factors from the rings that alter the relationship between the exposure and the population effect, while GEEs infer the population-averaged effect across all rings. As we expect there will be variance between rings and may want to explore it further, GLMMs are preferred over GEEs for this study. GAMs add together the non-parametric and parametric fits of separate regressors into a transformed regression. In this study, GAMs may be used if the observed relationship between delay to response and incidence offers a better fit than a purely parametric GLMM model. Regardless of model choice, the effect estimates should be similar and unbiased.

2. **…in the section of the stochastic transmission model (Line 209), the authors set an assumption about CATI implementation cost and population-based coverage. What are the reasons for not using a normal distribution simulation in the experiment? Similar to the conclusion of delay to CATI in Fig.2, what impact will it have on the results to reduce the required time of CATI and cover more people? It was necessary to try to simulate different situations about the population-based coverage to consider different action.**

Thank you for this comment regarding the fixed values of the duration of implementation and population-based coverage. In a sensitivity analysis, we ran the simulation for a range of values for the duration of implementation (optimistic=1 day, pessimistic=2 days) and the population-based coverage (optimistic=80%, pessimistic=50%, 60%, 75%). These ranges are based on the CATIs carried out in Juba, South Sudan in 2015 and Kribi, Cameroon in 2020.^1 2^ We updated the methods, findings, and discussion. We also added a table entitled *Power estimates from main simulations and sensitivity analyses* on page 17 and a section displaying graphs for each sensitivity analysis in the Supporting Information File*.*

**[Page 11, lines 225-8]:** We conducted two sensitivity analyses to explore the main assumptions of rapid 1-day duration of implementation and population coverage of 80%. We evaluated a longer duration of implementation of 2 days and lower population-based coverage estimates of 50%, 60%, and 75%, based on findings from field studies.^1 2^

**[Page 15, lines 295-300]:** Sensitivity analyses: Applying a slower duration of implementation of 2 days meant that 80% power is reached with >125 rings. Using lower population coverage of 50% and 60% increased the sample size required to >100 rings to reach 80% power. Lowering slightly the population coverage to 75% resulted in 79.1% power reached with 100 rings, which is close to the target of 80% power.

**[Page 17, lines 325-7]:** Based on an R_0 of 2.0, the sample size required to reach 80% power was 80—100 rings, which was generally maintained when population coverage reduced from 80% to 75%.

**[Page 18, lines 363-7]:** A duration of implementation of a single day has been shown in Cameroon and South Sudan[19, 20], but may not be sufficient to cover the entire ring. This potentially leads to an overestimation of the effect, with the sensitivity analysis finding higher sample size requirements

3. **Finally, as the author mentioned, some key variables are not included by the stochastic model, and I realized it is an obstacle that is hard be overcome, it does lower the strength of the analysis.**

Thank you for this observation. As mentioned, we agree that the simple approach to the simulation is limited by the inability to account for key variables (and potential confounders) that we would have to account for in the real-world prospective observational epidemiology study. Please note that we have already captured this discussion point page 17-9, lines 331-4.

**Reviewer 2 comments:**

4. The manuscript is well written and it has been performed with an adequate methodology. The results support the conclusions.

Thank you for this positive review.

**References**

1. Ouamba JP, Peyraud N, Mbarga NF, et al. Case-area targeted interventions for cholera control: experience from tail of cholera outbreak in Kribi, Cameroon. MSF Scientific Days. London, UK, 2021.

2. Parker LA, Rumunu J, Jamet C, et al. Neighborhood-targeted and case-triggered use of a single dose of oral cholera vaccine in an urban setting: Feasibility and vaccine coverage. *PLoS Negl Trop Dis* 2017;11(6):e0005652. doi: 10.1371/journal.pntd.0005652
